# Major Causes of Calf and Lamb Mortality and Morbidity and Associated Risk Factors in the Mixed Crop-Livestock Production System in Jamma District, South Wollo, Ethiopia

**DOI:** 10.1155/2021/6689154

**Published:** 2021-08-24

**Authors:** Atsbha Hadgu, Alemayehu Lemma, Tefera Yilma, Haben Fesseha

**Affiliations:** ^1^College of Veterinary Medicine and Agriculture, Addis Ababa University, Bishoftu, Ethiopia; ^2^School of Veterinary Medicine, Wolaita Sodo University, Wolaita Sodo, Ethiopia

## Abstract

Lamb and calf preweaning mortality and morbidity account for serious losses in sheep and cattle production and are, thus, a major factor in reducing profitability and adversely affecting the sheep and cattle farming. Thus, a prospective cohort study was conducted in Jamma district, Amhara Regional State, to determine the major cause of calf and lamb morbidity and mortality and associated risk factors. A semi-structured questionnaire and clinical assessment of the animals were conducted from 150 households to assess the potential risk factors. Besides, a total of 102 (81 fecal samples and 21 skin scrapings) were collected from 150 clinically ill and suspected animals to identify the cause of morbidity and mortality. The test of difference and correlation between variables were computed using chi-square and generalized linear model analysis. The total morbidity and mortality in calves were 33.3% and 2%, respectively, whereas for lamb, they were 27.3% and 32.5%, respectively. In calf, septicemia (100%) was a major cause of mortality, and diarrhea (54.6%) was the leading cause of calf morbidity followed by skin disease (30.1%), respiratory problems (12%), and septicemia (3.3%). Malnutrition was the most common problem in lambs causing up to 31.3% mortality followed by diarrhea 24% and respiratory problems 21.3%. The presence of a disease in adult cattle was significantly correlated to the presence of disease in calves (*p* < 0.001; *r* = 0.35). There was also a significantly higher positive correlation between sickness in adult sheep and lambs (*p* < 0.001, *r* = 0.45). Gastrointestinal parasitosis was identified in 82.7% of the samples collected from diarrheic and suspected calves (87.1% positive) and lambs (80% positive). *Monezia* species in lamb (33.3%) and *Coccidia* species in the calf (35.9%) had the largest morbidity rate. *Ctenocephalides canis* (16.7%) and *Linognatus* species (41.7%) were common ectoparasites identified in calves, while *Melophagus ovinus* was the only ectoparasite of lambs recovered (62.5%). In conclusion, the high morbidity found in calves and morbidity and mortality in lambs are known to seriously reduce the profitability of the smallholder cattle and sheep production in the area by affecting the availability of replacement animals and causing a detrimental effect on herd expansion and productivity. In further studies, establishing the specific causative agents, control of diseases in the adult, and improvement in feed resources should be the major areas that need to be considered to mitigate calf and lamb morbidity and mortality currently affecting the area.

## 1. Introduction

Livestock as an integral part of Ethiopian agriculture contributes considerably to sustainable food security and poverty reduction in the country. Over 85% of the subsistent smallholder farmers and almost all pastoralists depend on livestock as a major economic activity for their livelihoods [1, 2]. The contribution of livestock to the national economy accounts for about 19% of GDP and 20% of export earnings and can be explained in terms of food production, the supply of inputs and services for crop production, the raw material for agroindustry, cash income, and export earnings and investment and plays a role as a generator of employment [3, 4]. Moreover, the livestock sector supports and sustains enterprises and groups linked and associated with the livestock value chains, including the livestock traders, transporters, slaughter processors, feed manufacturers, and veterinary drug suppliers [5].

Ethiopia has the largest livestock inventories in Africa, estimated at 59.5 million cattle and 30.70 million sheep [3]. The small ruminant population of Ethiopia is one of the largest in Africa [6]. Most of the small ruminant population of the country is kept by smallholder farmers, and small ruminant production in the country is basically traditional [7]. However, the current contribution of the livestock subsector in Ethiopia is below its potential [8], and it is mainly due to the low genetic quality of local breeds, poor nutrition, animal health problems, poor husbandry, and poor infrastructure of the livestock sector of the country [9, 10].

The livestock is characterized by high mortality. A retrospective study undertaken in 2015/16 in major livestock production systems of Ethiopia reported alarmingly high annual losses of young stock from birth-to-weaning age and premature losses in terms of abortion and stillbirth. The mean annual birth-to-weaning mortality in the mixed crop-livestock system was reported in the range of 9.2–14% in calves, 14.9–33.5% in lambs, and 17.6–24% in kids, and the premature losses in terms of abortion and stillbirth were 3.0–8.7% in cattle, 7.5–8% in sheep, and 9.3–14.4% in goats [4, 11]. The annual direct losses from ruminant mortality are generally estimated at 8–10% of the cattle herd and 14–16% of the sheep flock. Youngstock mortalities constitute the larger share of constraints to herd expansion and genetic improvement [12].

Young animal diseases that cause morbidity and mortality are the results of the complex interaction of the management practices, the environment, infectious agents, and the animal itself [13]. Different management and environmental factors such as colostral feeding, housing, calving assistance, production system, herd size, season, and hygiene of microenvironment were reported to affect significantly calf morbidity and mortality [14]. Mortality of neonates of ruminants was mainly attributed to conditions such as diarrhea and pneumonia [13], joint problems, umbilical diseases, trauma, congenital abnormalities, nutritional deficiencies, dystocia, and other infections [15, 16] associated with poor housing, hygiene and nutrition [13], bovine viral diarrhea virus in pastoral, periurban, and mixed crop-livestock farms in central and Northwest Ethiopia [17]. Also, the incidence of calf morbidity among under six months of age was 34.1%, and that is due to calf diarrhea, pneumonia, septicemia, dehydration, and navel illness, in the Siyadeber and Wayu districts of Amhara Region, Ethiopia [18].

Lamb mortality accounts for serious losses in sheep production and is, thus, a major factor in reducing the profitability of sheep farming. One of the most important production factors that adversely affect small ruminant production is the high preweaning mortality of young lambs. Studies indicate that up to 50% of the lambs born can die, mainly due to diseases and other causes such as adaptation failure, dystocia, cold stress, starvation, and mismothering [19].

The success of any breeding program, as well as the future of the smallholder dairy farms, depends upon the rate of survival of calf crops produced. Accordingly, calf morbidity and mortality are of great concern for the dairyman [20, 21]. The shortage of dairy replacement heifers is one of the major hindrances to the development of smallholder dairy production in developing countries [22]. The current Ethiopian livestock breeding policy emphasizes upgrading the genetic makeup of the local stock through crossing with high-grade exotic breeds of cattle, sheep, goats, and poultry. As a result, the proportion of crossbred young stock is gradually increasing in the smallholder farms, mainly in the highlands of the country, suggesting a susceptible population that will need improved health and proper management [23].

Developing efficient livestock production that could reduce losses of young stock is important for farmers and pastorals to realize maximum benefits from their livestock resources. On the other hand, young stock mortality is one of the country-specific priorities that should be addressed. Such priority requires an informed decision with objective data. Although few studies have been conducted, still more remain to be carried out to determine various causes and risk factors to the calf and lamb mortality in one of the most common production systems, the mixed crop-livestock production system. Hence, this scenario aimed to identify production-specific causes and predisposing risk factors of calf and lamb morbidity. Based on the literature and personal observations from need assessments, it is possible to hypothesize that the management practices in livestock agriculture are poorly practiced resulting in high densities of livestock disease burden, especially in calves and lambs.

## 2. Materials and Methods

### 2.1. Study Area

The study was conducted in selected sites or peasant associations of Jamma district, South Wollo, Ethiopia. Jamma is one of the districts in the Amhara Region of Ethiopia which is located in the South Wollo Administrative Zone, which is 262 km northeast from Addis Ababa. Geographically, it is located between 10°06′24″ to 10°35′45″N and between 39°04′04″ to 39°23′03″E. The altitude range is between 1428 m and 2752 m above sea level. The district has 77.1% highland (Dega), 22.3% midaltitude (Woyina Dega), and 0.6% lowland (Kola) with an average annual rainfall of 1130 mm [24].

The livelihood of the people in this district is primarily a traditional smallholder mixed crop-livestock production system. The primary source of feed for both cattle and sheep is communal grazing with few households probably providing a supplement to a selected group of animals (pregnant and milking cattle). Straw and hay feedings are common both in cattle and sheep. The main source of water is a river where animals are watered almost every day [11]. The overwhelming majority of cattle and all sheep are local breeds. Few Holstein ∗ Zebu crosses are emanating from the National Cross-Breeding Program. Veterinary service is provided by the Veterinary Department of the Ministry of livestock and Fisheries. Artificial Insemination (AI) service is available for cattle only with infrequent and unorganized coverage mostly provided as a subsidiary to nearby cites, Dessie or Kombolcha.

### 2.2. Study Population

The study populations were farmers found in selected districts of Jamma, Amhara Regional State. A total of 150 smallholder farmers were randomly selected from three kebeles (kebele 09, kebele 11, and kebele 12), that is, 50 smallholder farmers from each kebele or peasant association (PA). All cattle and sheep including calves and lambs, respectively, were considered in this study. The age of animals was determined based on available birth files and dentition which will develop eight temporary incisors during the first month of age. The study animals were selected based on the following inclusion and exclusion criteria.

#### 2.2.1. Inclusion Criteria

Clinically healthy calves and lambs less than one year of age that were managed under a traditional smallholder mixed crop-livestock production system, upon detailed physical and clinical examination, were included in the current study. Moreover, those animals that were kept under extensive and semi-intensive were included in the study.

#### 2.2.2. Exclusion Criteria

All calves and lambs greater than one year of age were excluded from the study. Besides, those calves and lambs that were kept under intensive and that were not managed under mixed crop-livestock as well as calves that were not born in selected Kebeles (peasant association) were not included in the study.

### 2.3. Study Design

A longitudinal cohort study was carried out in the Jamma district of South Wollo in cattle and sheep to determine the major cause of calf and lamb morbidity and mortality and associated risk factors in the mixed livestock-crop production system. Data were collected for six months from November 2017 to April 2018.

### 2.4. Sample Size Determination and Sampling Method

The number of participants or households from each district was determined using the formula of Arsham [25].

(1) $$\begin{array}{c} N=\frac{0.25}{{\text{SE}}^{2}}, \end{array}$$

where SE is the standard error.

By considering the standard error of 0.05 with 95% coefficient interval, *N* = 0.25/0.05^2^ = 100. Thus, the total sample size was increased to 150 to increase the precision of the study.

Additionally, a purposive sampling technique was employed to select the study kebeles or peasant associations (PAs) from a total of 22 Kebeles. Thus, three kebeles or peasant associations (PAs) were selected based on accessibility, the presence of a sufficient number of target animals, the livestock-crop mixed production system, the animal owner's previous experience in extension programs, and the owner's willingness to participate in the study. A total of 50 volunteer livestock owners or households from each selected kebeles that fulfill the inclusion criteria were considered during the sampling period.

**Figure 1:**
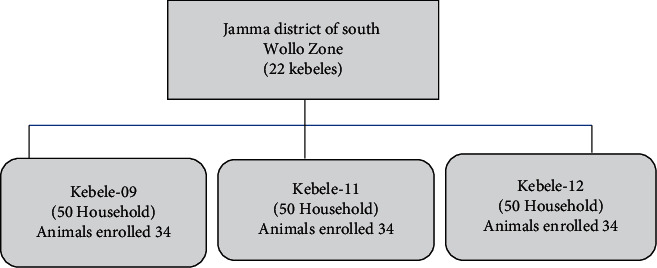
Graphic illustration of the sampling procedure for selecting study animals from Jamma district, South Wollo zone, Amhara Regional State, Ethiopia, 2019.

To study the potential risk factors for lamb and calf mortality and morbidity, Epi info. V. 7, software was used to determine the sample size for a cohort study which was used with an assumption of two-sided confidence level (1-alpha) (95%), power (chance of detecting) (80%), the ratio of exposed (with the specific factor) to the nonexposed (without the factor of interest) (1), the hypothetical proportion of morbidity with exposure, and hypothetical proportion of morbidity with nonexposure from previous research findings of the work of Ferede et al. [26], Megersa et al. [27], and Mohammed et al. [18]. Then, the calculated sample size with the aid of Fleiss with continuity correction was considered, and the largest sample measurement was considered for this study, that is, a total of 150 study animals from 150 households. Then, the sample size was proportionally allocated for the three kebeles (kebele 09, kebele 11, and kebele 12). However, a total of 102 lambs and calves that fulfilled the inclusion criteria were enrolled in the study (Figure 1).

### 2.5. Data Collection Method

The study was participatory, and animal owners (both men and women) and animal attendants have participated in the identification of causes and assessment of calf and lamb mortality and morbidity and related constraints having a negative impact on the productivity of livestock. Verbal consent was obtained from the livestock owners during sampling, the purpose of the study was briefly explained, and their willingness to participate in the study was obtained. Fecal and skin scraping samples were taken from diseased and susceptible calf and lamb of less than one year.

#### 2.5.1. Questionnaire Survey

A pretested structured questionnaire was prepared, and a total of 150 cattle- and sheep-owning farmers living in three nominated kebele or peasant associations were selected to face to face interviews. Questions included owner's information, livestock inventory, grazing, and aspects of management conditions such as feeding, housing, and breeding, management of calves and lambs, major health problem of adult and young animals, the presence of reproductive health problems, birth records of calves and lambs. In addition to this, information on a retrospective disease history, mortalities among the flock, and veterinary diagnostic, treatment, and control options were also gathered. Records of potential risk factors contributing to the occurrence of diseases were also carried out.

#### 2.5.2. Clinical Examination and Observational Study

During a house-to-house visit, animals with apparent signs of ailment were subject to a general clinical examination. A system-by-system approach of examination of the body organs of sick young and adult individual animals was conducted. Vital signs (rectal temperature, heart rate, respiratory rate, the color of mucous membranes, and palpation of superficial lymph nodes) and other overt clinical signs (coughing (up on cough induction), rumen movements (where applicable), skin condition, joints, and feet examination, depressed mentation, poor suckle reflex, weakness, and recumbence) found during the examination were recorded on a predesigned format. Due emphasis was given to young animals (calves and lambs), particularly when signs of diarrhea or pneumonia were observed.

In addition, a regular examination of the umbilicus was made in lateral recumbence to perceive an umbilical infection by means of rolling the 3 center fingers of every hand ventrally off the abdominal musculature for any evidence of growth or pain. The skin elasticity was once also assessed via pinching a fold of skin over the lateral side of the midneck region, rotating it 90 degrees, and finding out the size of time (in seconds) it takes for the fold to disappear to decide the extent of hydration. Moreover, the calves' health was evaluated via objective criteria of appetite, fecal consistency, hydration status, and behavior. After these detailed clinical and physical examinations, animals were categorized as “apparently healthy” or “diseased” [18, 28].

An observational survey was conducted on selected households for six months at a regular interval (once per week) to assess and monitor the incidence of calf and lamb mortality and morbidity. Data of the animal's feeding practice and bedding, including the farm health practices, were recorded in the designed format. Each clinical finding was also recorded in the separately designed format, but those animals that reached one year of age and above were excluded from the study even though they show morbidity signs during follow-up.

#### 2.5.3. Laboratory Confirmation of Selected Samples

A total of 102 samples (81 fecal and 21 skin scraping samples) were collected from 150 households selected from three kebeles of the study area. The study was participatory, and animal owners, both men and women, and animal attendants participated in the reporting of illness and mortality of calves and lambs. Fecal samples were collected directly from the rectum into a universal container. They were later on transported to the college of veterinary medicine of Addis Ababa University for laboratory analysis. The skin scraping was also collected into a screw-capped container in a 5% formalin solution. Similarly, skin scrapings were also analyzed at the parasitological laboratory.

Fecal samples were examined for the identification of internal parasite's eggs using direct smear, sedimentation, and floatation techniques (flotation fluid prepared from sugar, salt, and water in the proportion of 500 gram sugar and 400 gram salt dissolved in 1000 ml water). The skin scraping was mixed with a 10% sodium hydroxide solution to macerate the hair prohibiting examination [29, 30]. A reference parasite identification chart and pictures were used to identify the parasites [31–34].

### 2.6. Data Management and Statistical Analysis

All the data collected through the questionnaire and laboratory examinations were entered into a Microsoft Excel 2013 spreadsheet and transferred to SPSS Version 20.0 for statistical analysis. Variables such as morbidity and mortality were first described using means and proportions. Relationships were evaluated using Pearson's correlation. Test of difference between variables were computed using chi-square and Fisher's exact test. Bivariate logistic regression was computed to estimate the magnitude association between risk factors and the disease. Risk factors having a significant association with the disease were further analyzed by multivariate logistic regression analysis using a 95% confidence level (CI). The *p* value was held at less than 0.05 to define significant differences.

### 2.7. Ethical Consideration

Ethical approval for this research was obtained from the Addis Ababa University Research Ethics and Review Committee. Before collecting samples, the aim of the study was explained verbally by insuring as the study will not cause any harm and the participants are free to leave the study if they desire. Then, different samples were collected from their cattle, and strict hygienic measures were adopted.

## 3. Results

### 3.1. Herd Structure and Size

According to the present investigation, the households were totally dependent on the mixed livestock-crop production system. Besides, 80% of the respondents were females whereas 20% of them were males. The mean annual delivery rate of newborn calves and lambs was 0.67 (95% CI: 0.58–0.75) and 9.94 (95% CI: 8.89–10.98), respectively, whereas the mean annual death rate of newborn calves and lambs was 0.02 (95% CI: −0.0026–0.043) and 3.23 (95% CI: 2.59–3.88), respectively. In the present study, it was reported that the morbidity of lambs 2.71 (95% CI: 2.29–3.13) was higher than that of calf 0.22 (95% CI: 0.15–0.29) in the study sites (Table 1).

### 3.2. Major Causes of Mortality and Morbidity of Calves and Lambs

A total of 100 calves were born alive of which three calves died, and the mortality of calves for the district was 2%. Total calf morbidity was 33.3% with 48.0%, 36.0%, and 16.0% of kebele 12, kebele 11, and kebele 09, respectively. From 1491 newborn lambs, 485 (32.5%) lambs were dead with 32.6%, 34.4%, and 33% of kebele 12, kebele 11, and kebele 09, respectively. Overall lamb morbidity was 407 (27.2%) with 30.9%, 34.9%, and 27.7% morbidity prevalence of kebele 12, kebele 11, and kebele 09, respectively (Table 2).

Various causes were responsible for the mortality and morbidity of newborn animals, both in cattle and sheep. Major causes of calf morbidity were diarrhea, respiratory problem, septicemia, and skin disease (ectoparasite and other causes of skin lesion) contributed with 54.6%, 12%, 3.3%, and 30.1% of the total morbidity. Septicemia (100%) was the leading cause of death in calves (Table 3).

Malnutrition was the major cause of lamb mortality with a prevalence of 31% followed by diarrhea (24.0%), respiratory problem (21.3%), skin disease (such as sheep pox, orf, and others) (4.0%), septicemia (2%), and miscellaneous causes (3.5%). Diarrhea (44% of all morbidity) was the leading cause of lamb morbidity followed by respiratory disease (28.7%) and skin disease (26.6%) (Table 3).

**Figure 2:**
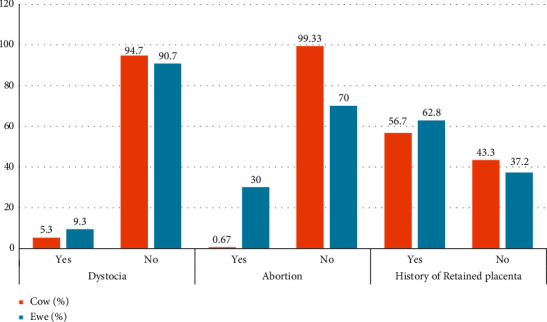
Proportion of different reproductive health problems in cow and ewe in the study district.

In the study villages, about 85 (56.7%) cows had a history of retained placenta and one cow had an abortion (0.67%). A total of 94 ewes have been aborted with 62.8% of the fetus with hair which indicated abortion happened in the last stage of pregnancy. Another 14 ewes had dystocia, while two had retention of the fetal membrane. Besides, 45 ewes had also a history of abortion (Figure 2).

### 3.3. Calf and Lamb Mortality- and Morbidity-Associated Risk Factors

The housing condition for cattle was generally rudimentary with no good ventilation. Newly acquired animals are not quarantined before introduction to the herd. Traditionally, many cattle owners 65 (43.3%) remove and throw the placenta into a river with a belief that this will improve milk letdown. Colostrum feeding is believed by the farmers to cause diarrhea and increase ectoparasite infestation; hence, only few farmers provide colostrum to their calves at the right time. Calves are allowed to suckle freely only after the time has passed for colostrum feeding. Mixed grazing of all ages of livestock is the major practice (80%). No additional care was known to be given to pregnant ewes and cows, and often, the dams themselves wean their young ones. Sick animals, particularly sheep, are not properly cared for and given special attention during pregnancy and parturition.

There were poor habits in the management of sick animals, especially sheep. There was no isolation of sick animals from the rest of the herds. They wait for their death, and some farmers also feed the mixture of the soil of the road dissolved in water for the animal with signs of diarrhea. There was no discrimination of young sheep from an adult during feeding, and they were fed with the same trough for all ages.

#### 3.3.1. Major Risk Factors Associated with Calf Mortality and Morbidity

Several risk factors were analyzed for their influence on calf morbidity and mortality. The major risk factors and morbidity and mortality prevalence were analyzed to determine the association between the risk factors and calf morbidity and mortality and summarized in Tables 4 and 5.

Keeping calves in a separate calving pen, group housing, colostrum feeding, age of the first supplementation, the presence of disease in adult cattle, and the average distance of the villages to the veterinary clinic were all significantly associated with both calf and lamb morbidity and mortality (Table 5; *p* < 0.05).

On the other hand, mixing all ages at communal grazing size, type of breeding, care given at calving, and opinion regarding colostrum were found to have very little association with calf morbidity and mortality. Some of the periparturient conditions were not directly associated with morbidity and mortality in calves (Table 5).

The presence of a disease in adult cattle was significantly correlated to the presence of disease in calves (*p* < 0.05; AOR = 0.092). There was a significant difference (*p* < 0.05) in calf mortality among the kebeles with almost all deaths occurring in kebele 2 (0.6 dead calve/household). The odds of calves becoming sick when adult animals are sick is significantly higher in kebele 1 and 2 (kebele 1- AOR = 0.042 and kebele 2–3.81; *p* < 0.05).

Providing separate calving areas, attended during the calving period, and age of feed supplementation were significantly associated with the presence of calf disease in the households (*p* < 0.05). But, the presence of communal grazing for all ages, calf housing system, veterinary care provided, type of breeding, practice of removal of placenta, time of first colostrum feeding, and type of feed supplementation were nonsignificantly associated (*p* > 0.05).

#### 3.3.2. Major Risk Factors Associated with Lamb Mortality and Morbidity

Provision of a separate lambing area, care at lambing, colostrum feeding within 12 hrs of birth, feed supplementation, age of the first supplementation, the presence of diseases in the adult, the average distance to the clinic, and veterinary care provided for the young were all significantly associated (*p* < 0.05) with lamb morbidity and mortality (Table 6).

The odds of lambs becoming sick when adult sheep are sick is significantly higher in all kebele's (kebele 1 AOR = 1.86, 95% CI; 1.086–3.18, kebele 2–21.81, and kebele 3–6.93; *p* < 0.05). There is a significantly higher positive correlation between sickness in adult sheep and lambs (*r* = 0.45, *p* ≤ 0.001). There was a significant difference (*p* < 0.05) in lamb mortality among kebeles with more deaths occurring in kebele 2 with 4.18 ± 0.55 dead lambs/household (kebele 1 = 1.94 ± 0.55; kebele 3 = 3.58 ± 0.55). Except for kebele, all the major risk factors (housing, colostrum feeding, supplementary feed, and age for supplementary feed) were associated with the presence of disease in lambs of the smallholders with a *p* value < 0.05.

All the major risk factors became significantly (*p* < 0.05) associated with the presence of disease in lambs. These were provision of lambing pen, attended during lambing, placenta removal practice, time of first colostrum feeding of lambs, feed supplement for lamb, age at which lambs started nonmilk feed, veterinary treatment provided, and the presence of disease in adult sheep. The factor that does not significantly associate (*p* > 0.05) with the presence of disease in lamb is the beliefs of owners regarding the importance of colostrum feeding (Table 7).

### 3.4. Laboratory Results

#### 3.4.1. Prevalence of Internal Parasites

From the total 81 fecal samples and 21 skin scrapings collected for identification of causal agents in calves and lambs, the prevalence of gastrointestinal parasitism was 82.7% (67/81) with 80% for samples from lambs and 87.1% for samples from calves. More than one parasite egg (a sign of mixed infection) was found in 28 samples (15 calves and 13 lambs). Different kinds of nematode, trematode, protozoa, and cestode parasite eggs were identified based on morphological structures (Table 8). Almost all risk factors (species, breed, sex, age, and village) considered were nonsignificantly (*p* > 0.05) associated with the prevalence of parasitic infestation in lambs and calves.

#### 3.4.2. Prevalence of External Parasites

**Figure 3:**
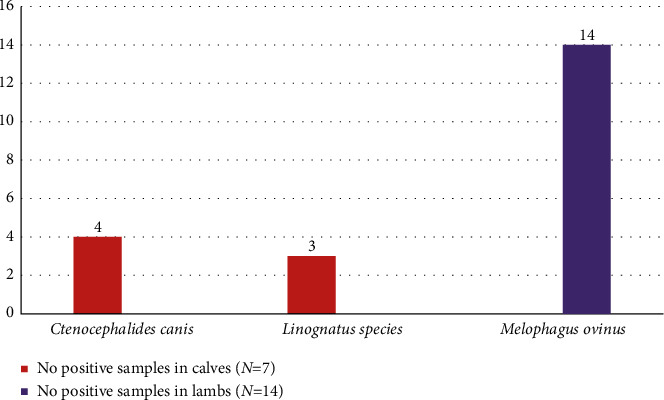
Frequency of ectoparasite species identified during the skin scrapping.

There were 66.7% positive skin scraping samples from lambs and 33.3% from samples in calves. *Ctenocephalides canis* and *Linognatus* species were most common in calves, while *Melophagus ovinus* was the only ectoparasite recovered in skin scraping samples from lambs. Species, sex, age, and kebele risk factors were not significantly related (*p* > 0.05) with the prevalence of ectoparasitic infestation in lambs and calves (Figure 3).

## 4. Discussion

This study was conducted to determine the prevalence and major causes of morbidity and mortality of calf and lamb and identify the influence of various risk factors on morbidity and mortality. Accordingly, the total morbidity and mortality in calves were 33.3% and 2%, respectively. Different authors reported a wider range of calf morbidity and mortality rates in Ethiopia. The mortality rate found in this investigation was lower than that in previous studies conducted by Asmare and Kiros [35], Wudu et al. [21], Ferede et al. [26], Romha [36], Megersa et al. [27], Yitagesu et al. [17], and Islam et al. [37] who reported 20%, 18%, 11.6%, 9.3%, 9.2%, and 6.29% mortality rates, respectively. But, it is comparable with the reports of Heinrichs and Radostits [38] who reported a 3% to 5% calf mortality rate and McNeil [39] who reported 3% in Australia and Lorenz et al. [40] who reported less than 6% in the UK. The mortality differences may be due to differences in herd and breed composition because many of the previous studies were conducted on crossbred dairy cows in smallholder farms. The herd composition in this study was dominated by local indigenous breed cattle, which are assumed to be less susceptible to diseases and environmental effects compared to crossbred calves. Furthermore, there were no fewer calves per household (maximum of two calves).

Morbidity comparisons are inconsistent as the results of the different reports were drawn by different approaches. Some involved advanced diagnostics techniques [17, 41], while others are obtained from interviews of livestock owners mostly relying on their ability to diagnose the diseases [4, 18, 21]. The morbidity rate of the calf in the present study was lower than that in the study of Asmare and Kiros [35] who stated 66.7% in Wolaita Sodo town and its suburbs, whereas this was comparable with the previous study of Mohammed et al. [18] who reported 34.1% in Siyadeber and Wayu district, North Shewa, Amhara, Ethiopia. On the other hand, the morbidity rate in the current study was higher than that in the study of Megersa et al. [27] who reported a 29.3% rate. On the other hand, it is lower than the 47.3% calf morbidity rate from a study in Bahir Dar by Ferede et al. [26]. This variation in prevalence might be due to the number of calves examined, herd-level risk factors, husbandry system, calf's age range, breed type, and agroecology. Previous studies conducted in Ethiopia revealed that most of the studies were conducted on government-owned farms and research institutes having large herd sizes comprising exotic and crossbreed calves, frequently known for higher calf morbidity [4, 11, 36].

Similar to the present findings, the studies by Mohammed et al. [18], Fentie et al. [4], Wudu et al. [21], and Fentie et al. [11] have identified the three most important disease problems in the young calves, diarrhea, septicemia, and pneumonia. Diarrhea was the most important cause of calf morbidity in this study similar to the one indicated by Mohammed et al. [18], Wudu et al. [21], and Asmare and Kiros [35]. However, the current finding of calf morbidity is higher in prevalence (54.7%) than that indicated by Wudu [42] (42.9%) and lower than 63.3% investigated by Asmare and Kiros [35]. Yitagesu et al. [17] reported that bovine viral diarrhea is also the cause of calf morbidity and mortality. Similarly, studies by Olsson et al. [43] in Swedish dairy herds, Sivula et al. [44] in Minnesota dairy heifer calves, and Debnath et al. [45] in smallholder traditional farms of Bangladesh all reported that diarrhea and pneumonia were the most prevalent calf disease [17, 18, 35]. On the contrary, Shiferaw et al. [14] reported that pneumonia was the major cause of calf morbidity in dairy farms of Holeta. The variation in the prevalence was multifactorial including variation in the time of colostrum feeding, hygiene of the barn, and handling of different feeding and drinking equipment [18, 35, 46].

The prevalence of respiratory problems corroborated with the report by Wale [47] who showed a 10.6% prevalence. The prevalence of respiratory problems (12.1%) and septicemia (3.1%) in the current research was lower than that in the report of Mohammed et al. [18] (23.9% and 19.6%) and Wudu et al. [21] (18.6% and 12.4%) and higher than the finding of Ferede et al. [26] (4.9% and 3.9%). Fentie et al. [4] reported a higher prevalence of respiratory syndrome (17%) as compared to our investigation. Calf skin disease (commonly ectoparasite) was found to have a prevalence of 30.6% which was higher than 12% by Ykealo [48] and lower than 50% by Mengesha et al. [46]. The difference may be attributed to the difference in the deworming program used and the degree of exposure, herd size, environmental stress, and stocking density [4, 11, 18].

Similar to the calf, lamb morbidity and mortality and their major causes were also identified. In this investigation lamb mortality and morbidity were 32.5% and 27.2%, respectively. This mortality rate of the current research is higher than that in the report of Khan et al. [49] who reported 21.4% in Pakistan and Tifashe et al. [50] who stated 7.04% in Wolaita Sodo Zuriya district, Southern Ethiopia. However, this was lower than that in the report of Woldemariam et al. [51] who reported a 40% lamb mortality rate in the Ebinat district of the Amhara Regional State and Bekele et al. [52] who stated mortality rates of 72.9% on farm and 71.8% on station in highland sheep of Ethiopia. Besides, it also reported morbidity rates of 88.4% on the farm level of the same area. Raghavendra et al. [53] stated different reports of lamb mortality in different sites of Andhra Pradesh parts of India, that is, 58.9% in Anantapur district, 19.35% in Chittoor, 11.96% in Kadapa, and 9.79% in Kurnool. The prevalence of morbidity in this study was comparable with that in the work of Khan et al. [49] who reported 31% in lambs of Pakistan. However, Tifashe et al. [50] reported a morbidity rate of 22.27% in lamb of Wolaita Sodo Zuriya district, Southern Ethiopia. The variation in disease and death rate might be due to feeding practice, agroecological difference, and farm husbandry practice in the study sites of the other studies. Furthermore, some of these investigations might have focused on farms with a high report of mortality rate [49, 54].

Malnutrition was the most common dominant cause of lamb mortality. Diarrhea and pneumonia were the most commonly found causes of lamb mortality that also agree with the work of Woldemariam et al. [51] which showed they were the most common problems next to malnutrition as represented by 31.3% of the total mortality. Diarrhea and pneumonia contributed 23.9% and 21.3 of the total mortality, but this finding does not agree with Woldemariam et al. [51] and Mukasaa-Mugerwa et al. [55]. Woldemariam et al. reported that diarrhea has a 51% of the total mortality in their study at Ebinat district, Amhara Regional State, while Mukasaa-Mugerwa et al.'s [55] finding was a 54% respiratory case as the most common cause of mortality. This may be due to variation in animal husbandry practice and disease prevalence [4, 11, 18, 35].

Skin disease (sheep pox, orf, and others), poor mothering immediately after parturition, and predators were also causes of lamb mortality with 12.8%, 1.2%, and 1.9% of the total lambs' mortality. Even though lamb morbidity is a result of complex interactions of different risk factors, there are diseases that cause lamb illnesses. Diarrhea was the major most common cause of lamb morbidity with a prevalence of 44% followed by respiratory problems, skin disease, fasciolosis, and septicemia (28.7%, 26.6%, 0.7%, and 0.7%, respectively). Similarly, Tifashe et al. [50] stated respiratory infections as a major cause of lamb morbidity in Wolaita Sodo district and Khan et al. [49] stated that pneumonia (47%) and diarrhea (25.3%) were the major factors for morbidity; in contrast to the current study, pneumonia (55.1%) and diarrhea (27%) were the main causes of mortality in lambs of Pakistan. Also, Woldemariam et al. [51] reported that diarrhea (51.0%) and pneumonia (38.5%) were the main causes of mortality in Ebinat woreda of Amhara National Regional State, Northwestern Ethiopia. On the other hand, Bekele et al. [52] stated that nutritional and managemental factors were accountable for mortalities in lambs whereas fascioliasis, ectoparasites, and nasal myiasis were the main causes of morbidity and mortality in lambs. The variation in the frequency of the distribution of the cases of lamb in different areas and the age of lambs was considered since some studies showed lamb below six months of age. The lamb mortality due to different infectious diseases might be attributed to poor nursing care and the primiparous dams, that led to less production, low-quality colostrum, and low level of immunoglobulin transfer [56].

Major risk factors for calf mortality and morbidity were analyzed to assess the significant association with calf mortality and morbidity. Care for pregnant cows such as providing separate calving pens and good hygienic practices were important to maintain the health of the calves in good status. The presence of a separate calving pen and the availability of veterinary services nearby had a direct influence on this study. Calves that become sick have the chance to visit the nearby veterinary clinic, and hence, the chance of survival is higher than that of those that were located far and did not have the chance to go to the clinic earlier in the course of their illness. Similarly, Fentie et al. [4] reported that the provision of health services and improving health management have a great impact in reducing calf morbidity and mortality against different diseases. Good housing hygiene, biosecurity, and proper colostrum feeding help to increase resistance against different infections and reduce calf morbidity and mortality [18, 21, 57].

The presence of disease in adult cattle and calves receiving colostrum within 12 hours of birth is significantly associated with calf mortality and morbidity. As Drewry et al. [58] reported, ingestion and absorption of enough quantity and quality of colostrum is a critical determinant for the health and survival of neonatal calves; calves that did not receive adequate colostrum are shown to have a higher overall death rate. To ensure adequate protection against disease, calves rely on the intake of an adequate amount of quality colostrum within a few hours of birth [59]. The ability of the neonate to absorb immunoglobulin starts to decline progressively after 6 to 12 hours from birth [57]. Factors such as the age of nonmilk feed supplementation, the presence of disease in adults, and young cattle were significantly correlated with calf morbidity. There was a high correlation of sickness in adult cattle with morbidity of calves. This may be due to the presence of a high probability of contact of different ages of cattle in the grazing area as well as at home. Fentie et al. [4] also stated that farmers only offer poor-quality feed, mainly natural grass and dry crop residues, for their calves. Besides, these feed stuffs have a low level of nutritional value since they have low crude protein, mineral contents, and digestibility [60]. Fentie et al. [4] and Mohammed et al. [18] also reported provision of colostrum has significantly minimized the risk of calf mortality in a different part of Ethiopia. Hence, it is recommended to regularly allow the calf to properly suckle the colostrum from the dam within the first two days of postdelivery to have effective transfer of passive immunity and colostral immunoglobulins [61].

Provision of a separate lambing pen, attending ewes at lambing and provision of colostrum within 12 hours of birth, and early supplementation of the young have all contributed to the reduction of lamb mortality. Although these are practiced, the mortality remained relatively higher compared to reports elsewhere. But comparatively, it is lower than the 40% prevalence previously reported in the Ebinat woreda of Amhara National Regional State, Northwestern Ethiopia [51].

Various clinical signs exhibited by the lambs suffering from diarrhea included profuse watery diarrhea with often loose but scanty feces which were occasionally mixed with blood. These animals showed anorexia, weakness, dullness, and depression with subnormal temperatures [51]. Diarrhea is associated with mild to severe dehydration and leads to loss of electrolytes. The animals with this stage need electrolyte therapy [62] but there was a limitation of veterinary clinic access to some kebeles of the woreda. Gulliksen et al. [63] noted that calf diarrhea was a major ailment and the predisposing factors were mainly the lack of colostrum and failure to absorb cloistral antibodies. Diarrhea is caused by different infectious and noninfectious agents [64], and there are different GIT parasites causing diarrhea. Age was not related to parasitic load in this study although helminth prevalence is known to increase with age [65]. GIT parasite burden and diversity increase with age and at weaning and end of the first year of life, and calves acquired the parasite spectrum similar to that of adult cattle. According to Wymann et al. [65], as age increases, calves are given fresh grass as supplemental feed. However, this is different from the farms studied in the periurban areas of Bamako in Mali having different management systems.

*Melophagus* was ectoparasite of lamb identified in Jamma. *Melophagus ovinus* is common hematophagous ectoparasites of sheep [66], and it is a vector for vector-borne diseases such as *Trypanosoma melophagium* [67], *Anaplasma ovis*, *Acinetobacter* [68], and *Borrelia burgdorferi* [69]. There is a probability of the presence of these vector-borne diseases in Jamma.

As James [70] noted, young animals are more susceptible to ectoparasite infestations, similar to the present finding, partly because of a higher proportion of accessible surface-to-body volume, poor grooming behavior of young animals, and their immature immunity. To reduce the negative impact of calf mortality and morbidity on livestock production, additional study and appropriate measures should be carried out and implemented.

The limitation of this study was that it does not determine the bacterial causes of mortality and morbidity in both calves and lambs. Besides, this research work was conducted with a smaller sample size, smaller area coverage, and in a limited period of the year which make it difficult to include all the potential risk factors for mortality and morbidity.

## 5. Conclusions

Lamb and calf mortality and morbidity are recognized to be major constraints of productivity in the study area. The major risk factors identified are the presence of diseases in the adult animal, improper colostrum feeding, and feed shortages. Lambs and claves growing in households, where there is a sick adult animal, have been found to be more prone to contract the disease and die of the same diseases compared to those growing with healthy adults. The common practice of communal grazing and housing of animals of all ages seem to have contributed to the transmission of the diseases. Septicemia and diarrhea were known to be the major causes of mortality and morbidity in calves, while malnutrition, diarrhea, and respiratory illnesses were the major causes of mortality in lambs. Similar to recent surveys in other parts of Ethiopia, diarrhea and respiratory illness can be generally considered to be the major causes of morbidity and mortality in both animals. Young animals were generally given little attention including adequate colostrum feeding, which also adds to the problem. This condition has been known to be from lack of awareness although the correlation between lamb and calf mortality, and knowledge of colostrum feeding by owners has not been established with the present data. Unless there are mechanisms that are devised to create awareness, reduce the risk of disease, and improve feeding management, the trend of morbidity and mortality in both calves and lambs can be considered alarming. In conclusion, further detailed investigations should be performed to specifically establish the magnitude of the problem and identify all causes of sickness and death. Appropriate young stock mortality and morbidity reduction packages should be designed and implemented to reduce calf and lamb mortality and morbidity. Awareness should be created for the livestock owners regarding husbandry practices that can reduce the loss of the youngstock. Proper veterinary service and disease identification mechanisms have to be designed and implemented. Thus, all these can help to reduce the different risk factors and minimize their detrimental effect.

## Figures and Tables

**Table 1:** Mean annual calf and lamb delivery, morbidity, and mortality distribution in the study area (*N* = 150).

Variables	Mean	Std. deviation	Minimum	Maximum	95% CI (lower–upper)
No. of newborn calves	0.67	0.54	0.0	2.0	0.58–0.75
No. of sick calves	0.22	0.43	0.0	2.0	0.15–0.29
No. of dead calves	0.02	0.14	0.0	1.0	−0.0026–0.043
No. of newborn lambs	9.94	6.47	0.0	32.0	8.89–10.98
No. of sick lambs	2.71	2.59	0.0	10.0	2.29–3.13
No. of dead lambs	3.23	3.99	0.0	17.0	2.59–3.88

**Table 2:** Distribution of mortality and morbidity of newborn calf and lamb across the study villages.

Determinant factors	Category	Number of calves	Proportion (%)	Number of lambs	Frequency (%)
Mortality of newborn	Kebele 09	0	0.0	160	33
	Kebele 11	3	8.1	167	33.4
	Kebele 12	0	0.0	158	32.6

Morbidity of newborn	Kebele 09	8	16.0	139	34.2
	Kebele 11	18	36.0	142	34.9
	Kebele 12	24	48.0	126	30.9

**Table 3:** Causes of lamb and calf morbidity and mortality in the mixed livestock-crop production system.

Causes	Category	Mortality		Morbidity	
		Frequency	Proportion (%)	Frequency	Proportion (%)
Malnutrition	Lamb	47	31.3	9	6.0
	Calf	3	2.0	2	1.3

Diarrhea	Lamb	36	24.0	66	44.0
	Calf	8	5.3	82	54.6

Respiratory problem	Lamb	32	21.3	43	28.7
	Calf	8	5.3	18	12.1

Septicemia	Lamb	3	2.0	1	0.7
	Calf	100	100	5	3.3

Skin disease	Lamb	6	4.0	40	26.6
	Calf	3	2.0	46	30.6

**Table 4:** Effect of management risk factors on morbidity and mortality of calves.

Risk factors	Categories	*N*	Morbidity	*p* value	Mortality	*p* value
Communal grazing for all ages	Yes	76	27 (35.5%)	0.06	2 (2.6%)	0.09
	No	24	6 (25%)		1 (4.2%)	

Housing	Separate	64	20 (31.2%)	0.04	2 (3.1%)	0.06
	Group	36	13 (36.1%)		1 (2.8%)	

Age of supplementation	<3 weeks	30	7 (23.3%)	0.020	0	0.03
	>3 weeks	70	26 (37.1%)		3 (4.3%)	

Presence of disease in adult cattle	Yes	47	24 (51%)	≤0.001	2 (4.3%)	0.04
	No	53	9 (17%)		1 (1.9%)	

Presence of young stock disease	Yes	35	33 (94.3%)	≤0.001	3 (8.6%)	0.002
	No	65	0 (%)		0	

**Table 5:** Effect of periparturient calf management on morbidity and mortality of calves.

Risk factors	Categories	*N*	Morbidity	*p* value	Mortality	*p* value
Received colostrum within 12 hours of birth	Yes	96	30 (31.2%)	0.066	2 (2%)	0.07
	No	4	3 (75%)		1 (25%)	

Attended during calving	Yes	80	25 (31.3%)	0.93	2 (2.5%)	0.06
	No	20	8 (40%)		1 (5%)	

Calf with a separate calving pen	Yes	75	25 (33.3%)	0.05	0	0.003
	No	25	8 (32%)		3 (12%)	

**Table 6:** Association of lamb management with mortality and morbidity.

Risk factors	Categories	*N*	Mortality (%)	*p* value	Morbidity (%)	*p* value
Lambing pen	Separate	141	11.3	0.001	28.4	0.012
	Share housing with others livestock	1350	34.7		27.2	

Attended during lambing	Yes	406	14	0.0001	25.4	0.003
	No	1085	39.4		28	

Opinion regarding colostrum feeding	Important	445	28.5	0.361	26.5	0.191
	Not important	1010	33.9		27.5	
	Do not know	36	44.4		30.5	

Received colostrum within 12 hours of birth	Yes	641	15.9	0.0001	29.6	0.002
	No	850	45.3		25.7	

Supplement for lamb	None	326	38.7	0.012	27.9	0.006
	Straw	813	35.4		30.4	
	Straw and hay	99	24.2		22 (22.2)	
	Concentrate	253	18.6		47 (18.6)	

Age of supplemented feed for lamb	<2 weeks	465	12.3	0.0001	150 (32.3)	0.002
	>2 weeks	809	47.8		210 (26)	
	Any time	217	18.9		47 (21.7)	

**Table 7:** Morbidity of lambs on different managemental factors in Jamma district.

Factors	Category	Frequency of lamb morbidity	Proportion (%)	Chi-square (*X*^2^)	*p* value
Veterinary care provided for sheep	Vaccine	0	0.0	22.71	0.0001
	Deworming	44	58.67		
	Vitamin supplementation	10	66.67		
	Hoof care	0	0.0		
	No	41	80.39		

Provision of separate lambing pen	Separate	11	52.38	10.76	0.005
	Share housing with other livestock	0	0.0		
	No	84	67.74		

Placenta removal practice	Incinerate	60	68.18	11.89	0.008
	Throw it away	34	61.82		
	Feed it to the dogs	0	0.0		
	Do nothing	1	100.0		

Beliefs of owners regarding colostrum feeding	Important	65	67.01	1.59	0.21
	Not important	30	56.60		

Attended during lambing	Yes	26	53.06	13.88	0.001
	No	69	71.88		

Time of first colostrum feeding of lambs	<12 hours	53	74.65	7.43	0.006
	>12 hours	42	53.16		

Feed supplement for lamb	None	20	76.92	16.69	0.002
	Straw	57	69.51		
	Straw and hay	4	4.44		
	Concentrate	14	51.85		

Age of supplemented feed for lamb	<2 weeks	47	79.66	12.31	0.002
	>2 weeks	13	44.83		
	Any time	35	56.45		

**Table 8:** List of helminths eggs identified during the fecal examination.

Species of parasites	No. positive samples in calves (*N* = 39)	No positive samples in lambs (*N* = 42)
*Chabertia* species	1	4
*Coccidia* species	5	1
*Fasciola* species	3	3
*Cooperia* species	0	1
*Haemonchus* species	1	0
*Monezia* species	0	5
*Ostertagia* species	1	2
*Toxocara* species	2	1
*Trichostrongylus* species	1	2
*Strongyle* species	1	0
*Trichuris* species	2	1
*Nematodirus* species	2	0

## Data Availability

The data will be provided upon request from the corresponding author.

## Abbreviations

AI: Artificial insemination
ANOVA: Analysis of variance
CI: Confidence interval
CSA: Central Statistics Agency
EARO: Ethiopian Agricultural Research Organization
GDP: Gross domestic product
GIT: Gastrointestinal tract
IBC: Institute of Biodiversity Conservation
IGAD: Intergovernmental Authority on Development
ILCA: International Livestock Center for Africa
MOA: Ministry of Agriculture
NABC: Netherland-African Business Council.

## Supplementary Materials

Supplementary Materials Additional file-1: questionnaire format prepared to assess calf and lamb mortality and morbidity and associated risk factors in Jamma district Additional file-2*:* vernacular name of common disease of sheep and cattle and figure of different ectoparasites in the Jamma district.
