# Supplementary material for: Major Causes of Calf and Lamb Mortality and Morbidity and Associated Risk Factors in the Mixed Crop-Livestock Production System in Jamma District, South Wollo, Ethiopia
Source: Vet Med Int. 2021 Aug 24;2021:6689154. doi: 10.1155/2021/6689154 (PMC8407976; doi:10.1155/2021/6689154)
Supplement: Supplementary Materials — Additional file-1: questionnaire format prepared to assess calf and lamb mortality and morbidity and associated risk factors in Jamma district Additional file-2: vernacular name of common disease of sheep and cattle and figure of different ectoparasites in the Jamma district. [file 6689154.f1.zip › 6689154.f1/Questionnaire for calf & lamb mortality & morbidity format.pdf]

Questionnaire for calf and lamb mortality and morbidity and associated risk factors data collection format

Interview performed by: __________ Questionnaire Number: ______________________

Please write NA if the question is not applicable, NR when the respondent decline to do so!

Part I. Owner's information

1. Owner's name (optional): __________________________
2. Gender: _ Male __Female
3. Address: Region/City: __________
4. District: ______
5. Kebele: __________
6. GPS coordinates (farm): Latitude: _________ Longitude: ______
7. Production system: ______ Peri-urban farm ______Mixed crop-livestock, _________Pastoral
8. Experience of farming: _________________________________

Part II. Farm information, facilities and personnel

9. Domestic livestock inventory during the past 12 months

Animal	Newborn (<1m)		Pre-weaning (1m-Wn)		Post-weaning (Wn-1yr)		Young (1-2yr)		Adult (>2yrs)		Total	
	M	F	M	F	M	F	M	F	M	F	M	F
Cattle												
Sheep												
Goat												
Camel												
Others												

Wn= Weaning

10. Do animals of all ages share the same grazing areas? (Include N^{o} of animals per unit area?)

	Cattle	Sheep
Yes, all herd grazes the same area at the same time		
Yes, all herd grazes the same area but at different times		
If so, is preference given to young animals? Circle one	Yes / No	Yes / No
No, there are different areas for different groups of animals		
If so, what ages graze together?		

11. Do animals graze for part of the day during the entire year?

Cattle: ___ Yes ___ No, it varies by season___ NR

Sheep: ___ Yes ___ No, it varies by season___ NR

12. Do animals of all ages share the same housing areas?

Cattle: ___ Yes ___ No ___ NR

If yes, what ages are housed together?

____

Sheep: ___ Yes ___ No ___ NR

If yes, what ages are housed together?

____

13. Describe the type of housing:

Animal	For adult	For young	Any bedding?
Cattle			
Sheep			

14. Do you clean/disinfect your farm/barn?

Animal	Frequency of cleaning Daily/weekly/monthly	How it is done Manure removal/spray/foot bath	What is used for cleaning Water/detergent/chemical
Cattle			
Sheep			

15. What is the source of replacement stock (select all that apply)?

Cattle: ___Self ___ neighbor ___ market___ other, specify _________________________

Sheep: ___Self ___ neighbor ___ market___ other, specify _________________________

16. Are newly acquired animals quarantined (housed separately from herd) before introduction to herd?

Cattle: ___ Yes ___ No ___ NR

Sheep: ___ Yes ___ No ___ NR

17. What is the source of the water for the animals? ____________________________

18. Number of animal attendants (include if there is specific training for care giving)

Species	Family Members	Hired	Total n^{o} of attendants
Cattle			
Sheep			
Goats			
Camel			

19. Do you keep records of your animals such as breeding dates, calving dates, disease events, etc.?

Species	Breeding Dates			Birth Dates			Disease events			Treatment days			Death dates		
	Yes	No	NR	Yes	No	NR	Yes	No	NR	Yes	No	NR	Yes	No	NR
Cattle															
Sheep															

Part III. Management of the Herd

20. Do you provide any kind of treatment to the herd to prevent disease?

If yes, please check all types of veterinary care provided (specify as months or years).

Animal	Type of treatment	How often?	Traditional	Vet. clinics
Sheep/Goat	___ Vaccines			
	___ Dewormers			
	___ Vitamins			
	___ Hoof care			
	___ Other, describe:			
Cattle	___ Vaccines			
	___ Dewormers			
	___ Vitamins			
	___ Hoof care			
	___ Other, describe:			

21. Do pregnant animals receive special management care?

Care	Ewe/Doe	Cow
Vaccination of Pregnant		
Extra feeding during last trimester		
Separate parturition pen		
Keeping around house (limiting log distance travel)		

22. Type of breeding performed in the farm:
Cattle: ___ Natural breeding ___ Artificial Insemination (AI)
If Artificial Insemination (AI),
What is the source of AI semen? ____
How many services are typically required for successful breeding? ____
How many cows got pregnant in the last year (regardless of later pregnancy loss? ____
How many ewes got pregnant in the last year (regardless of later pregnancy loss)? _____
How many does get pregnant in the last year (regardless of later pregnancy loss)? ____
How many she-camels got pregnant in the last year (regardless of later pregnancy loss)? ____

23. Are the dams separated from the herd when she is approaching parturition? (include if there is any bedding used if a separate house is used?)
Cattle: ___ Yes ___ No ___ NR
Sheep: ___ Yes ___ No ___ NR
24. Are births witnessed by an attendant? ___ Yes ___ No ___ NR
25. During births, how do you decide when it is time to help the dam to deliver the calf/lamb/kid (please describe by species if necessary)? ___ NR
Cattle:
____
Sheep: ____
26. How often do you clean the floor where dams give birth (specify in days or months):
Cattle: ______________________ NR ___
Small ruminants: ________________ NR ___
27. What do you do with placentas, fetal membranes and aborted fetuses? Please describe by species if necessary.

	Cattle	Sheep
Incinerate		
Throw it away		
Feed it to the dogs		
Other, specify		

Part IV. Management of Calves, Lambs and Kids

28. What kind of care do you give to the new born?

Animal	Naval treatment	Colostrum feeding	Body cleaning	Hoof trimming	Assist standing	Assist suckling	Keep warm
Calves							
Lambs							

29. Do you think it is important to feed colostrum to neonates?___ Yes ___ No ___ NR

30. Does every newborn receive colostrum in the first 24 hours?

	Calves	Lambs
Yes, within 12 hours after birth		
Yes, sometime during the first 24 hours after birth		
I do not know		
NR		

Include reason if reduced or not fed at all to any of the species above, ____________________________

31. Any type of additional feed used for newborn calves/kids/lambs & its source amount and frequency of feeding ____________________________

32. If tools and equipment are used to feed milk, do you clean those? _____; How? ________________

33. Amount and frequency of milk/milk replacer given daily to the following types of animals:
Weak calf: _____ L/ ____; Normal calf: ______L/ ______; Weak lamb/kid: _______ ml/ _______; Normal lamb/kid: _____ ml / ____; Weak camel calf: _____ L/ ____; Normal calf: ______L/ ______

34. What do you do if a young animal will not suckle or take a bottle? Please, describe by species: ___ NR

Calves	
Lambs	

35. When do you introduce feed different from milk/milk replacer? NR____

Calves	
Lambs	

36. What steps do you take to wean young stock? (i.e. complete separation from dam, fence-line weaning, etc.) Please specify by species if necessary: NR ____

Calves	
Lambs	

37. Type of feed and quantity given during the weaning? And after weaning:

	Cattle	Sheep
Grazing, specify hours of grazing		
Concentrates, specify type and quantity		
Fodder, specify type and quantity		
Others, specify type and quantity		

38. What do you feed during the dry season months? Please specify by species if necessary, NR ____

Calves	
Lambs	

39. During which months do you encounter feed shortages? Please specify _______________NR ___
40. Do you prepare or use any feed supplement (silage, mineral block?): ___ Yes ___ No___ NR for which animal? ____________________
41. How do you use a watering and feeding trough?
Cattle: ___ separate for each calf ___ one trough for all ___ other, specify __________
Sheep: ___ separate for each lamb ___ one trough for all ___ other, specify __________

42. How frequent are the calves/lambs/kids given access to water before weaning? ___ NR

	Rainy season			Dry season		
	3 times/day	2 times/day	1 time/day	More than 3 times/day	2 times/day	1 time/day
Cattle						
Sheep						

Part V. Health problems, prevention and control

43. Major health problems for the farm in adult animals in order of importance ___ NR

	Cattle	Sheep

FIRST cause of disease		
Average duration of symptoms prior to death		
Average age affected		
SECOND cause of disease		
Average duration of symptoms prior to death		
Average age affected		
THIRD cause of disease		
Average duration of symptoms prior to death		
Average age affected		

44. Disease or disease syndromes causing sickness and death of young animals in order of importance

	Calves (<2yr)	Lambs (<1yr)
FIRST cause of disease		
Average duration of symptoms prior to death and number of mortalities		
Number of sick		
SECOND cause of disease		
Average duration of symptoms prior to death		
Average age affected		
THIRD cause of disease		
Average duration of symptoms prior to death		
Average age affected		

45. Do you separate animals when they get sick? ___________________

46. What is the most common cause of sickness and number of sick calf and lamb
______________________________________Calves ______________________________ lambs
47. What do you do if a young animal has diarrhea? ________________________________
______________________________________Calves ______________________________ lambs

48. How many dams in your herd abort or have stillbirth (birth of dead offspring) in the last 12 months?
______ cows ______ sheep ___ NR
49. How many of the aborted fetus have hair (indication of abortion in late gestation)? ______ cows ______ sheep ___ NR
50. How many offspring were born alive in the last 12 months?
______ calves ______ lambs ___ NR
51. Of the ones born alive, how many of them became sick in the last 12 months?
______ calves ______ lambs ___ NR
52. Of the ones born alive, how many of them died in the last 12 months?
______ calves ______ lambs ___ NR
53. Of the dams that gave birth in the last 12 months, how many had dystocia (difficulty of birth)?
______ cows ______ sheep ___ NR
54. Of the dams that gave birth in the last 12 months, how many had retained placenta (12 hours after birth still had not expelled the placenta)?

______ cows ______ sheep ___ NR

55. of the dams that gave birth in the last 12 months how many had mastitis or closed teats (no colostrum or milk)?

______ cows ______ sheep ___ NR

56. Who do you primarily seek help from when you have a sick animal?

___ Veterinarian ___ extension officer ___ animal health worker/Para-vet ___ neighbor

___ No one ___ other, specify _________________________________ ____ NR

57. How far do you have to travel to seek treatment for your sick animals? _______ Km___ NR

58. Are you satisfied with the animal health services you have available? ___ Yes ___ No ___ NR

If no, what do you consider the most useful improvements to animal health for you as a livestock owner/keeper? Please specify in order of importance (i.e. training, disease recognition and treatment knowledge, vaccination programs, etc.).

First ___________________

Second _________________

Third __________________

Fourth __________________

NR __
