# Supplementary material for: Major Causes of Calf and Lamb Mortality and Morbidity and Associated Risk Factors in the Mixed Crop-Livestock Production System in Jamma District, South Wollo, Ethiopia
Source: Vet Med Int. 2021 Aug 24;2021:6689154. doi: 10.1155/2021/6689154 (PMC8407976; doi:10.1155/2021/6689154)
Supplement: Supplementary Materials — Additional file-1: questionnaire format prepared to assess calf and lamb mortality and morbidity and associated risk factors in Jamma district Additional file-2: vernacular name of common disease of sheep and cattle and figure of different ectoparasites in the Jamma district. [file 6689154.f1.zip › 6689154.f1/Vernacular name of common disease of sheep and cattle and Figure of different ectoparasites in the Jamma district (1).doc]

**Vernacular name of common disease of sheep and cattle in the selected sites of study district**

| **Common disease and signs** | **Vernacular name** |
| --- | --- |
| FMD | *Aftegir/ Africa beshta* |
| Blackleg | *Worch wogi* |
| Ovine pasteurellosis | *Gfaw* |
| Sheep pox | *Fntata* |
| Coughing | *Sal* |
| Fasciolosis | *Kulkult* |
| Diarrhea | *Ktsen/ tekmat* |
| Orf | *Afemendil* |
| Anthrax | *Abeba senga* |

**Figures of calf and lamb ectoparasite that were taken during laboratory investigation**

**Figure 1**: Sheep ectoparasite (Melophagus ovinus) recovered from Jamma, South Wollo

**Figure 2**: Calf lice (Linognatus spp) recovered from calves at Jamma, South Wollo
